# Folic acid deficiency enhanced microglial immune response via the Notch1/nuclear factor kappa B p65 pathway in hippocampus following rat brain I/R injury and BV2 cells

**DOI:** 10.1111/jcmm.14368

**Published:** 2019-05-13

**Authors:** Man Cheng, Liu Yang, Zhiping Dong, Mengying Wang, Yan Sun, Huan Liu, Xuan Wang, Na Sai, Guowei Huang, Xumei Zhang

**Affiliations:** ^1^ Department of Nutrition and Food Science, School of Public Health Tianjin Medical University Tianjin China; ^2^ Tianjin Key Laboratory of Environment Nutrition and Public Health Tianjin China; ^3^ School of Basic Medical Sciences Tianjin Medical University Tianjin China

**Keywords:** folic acid, hippocampus, inflammatory, ischaemia‐reperfusion, microglial, Notch pathway

## Abstract

Recent studies revealed that folic acid deficiency (FD) increased the likelihood of stroke and aggravated brain injury after focal cerebral ischaemia. The microglia‐mediated inflammatory response plays a crucial role in the complicated pathologies that lead to ischaemic brain injury. However, whether FD is involved in the activation of microglia and the neuroinflammation after experimental stroke and the underlying mechanism is still unclear. The aim of the present study was to assess whether FD modulates the Notch1/nuclear factor kappa B (NF‐κB) pathway and enhances microglial immune response in a rat middle cerebral artery occlusion‐reperfusion (MCAO) model and oxygen‐glucose deprivation (OGD)‐treated BV‐2 cells. Our results exhibited that FD worsened neuronal cell death and exaggerated microglia activation in the hippocampal CA1, CA3 and Dentate gyrus (DG) subregions after cerebral ischaemia/reperfusion. The hippocampal CA1 region was more sensitive to ischaemic injury and FD treatment. The protein expressions of proinflammatory cytokines such as tumour necrosis factor‐α, interleukin‐1β and interleukin‐6 were also augmented by FD treatment in microglial cells of the post‐ischaemic hippocampus and in vitro OGD‐stressed microglia model. Moreover, FD not only dramatically enhanced the protein expression levels of Notch1 and NF‐κB p65 but also promoted the phosphorylation of pIkBα and the nuclear translocation of NF‐κB p65. Blocking of Notch1 with N‐[N‐(3, 5‐difluorophenacetyl)‐l‐alanyl]‐S‐phenylglycine t‐butyl ester partly attenuated the nuclear translocation of NF‐κB p65 and the protein expression of neuroinflammatory cytokines in FD‐treated hypoxic BV‐2 microglia. These results suggested that Notch1/NF‐κB p65 pathway‐mediated microglial immune response may be a molecular mechanism underlying cerebral ischaemia‐reperfusion injury worsened by FD treatment.

## 1 INTRODUCTION

Stroke is a type of acute cerebrovascular neuropathology with a high rate of disability, mortality and morbidity. Ischaemic stroke accounts for approximately 85% of all strokes.^1^ Microglia are the primary resident immune cells in the brain. Microglial activation is an important component of the neuroinflammatory response to ischaemic stroke.^2^ It has been demonstrated that the activated microglia migrate to the infract area to perform phagocytic clearance of cellular debris.^3^ In this way, it can play a role in protecting neurons following cerebral ischaemia. However, overactivated microglia release amounts of proinflammatory cytokines and/or cytotoxic factor such as nitric oxide (NO), tumour necrosis factor‐α (TNF‐α), interleukin‐1beta (IL‐1β) and interleukin‐6 (IL‐6), which may cause injury to healthy neurons and result in progressive neuronal damage.^4^, ^5^, ^6^ Therefore, microglial activation has the dual role in promoting beneficial and detrimental effects on neurons. Modulation of microglial activation for therapeutic purposes might be realized via suppressing the deleterious effects of these cells.

Folic acid, a member of the vitamin B complex, has been proven to be tightly associated with central nervous system function and development.^7^ In adults, a compelling and extensive epidemiological literature suggested a relationship between inadequate status of folate and increased risk of neurodegenerative and cerebrovascular diseases.^8^ Folate deficiency and resultant hyperhomocysteinaemia are not only associated with increased stroke risk but also increased oxidative DNA damage and larger ischaemic injury volume after MCA occlusion/reperfusion.^2^, ^9^, ^10^ However, the exact mechanism of the effect of FD on neurologic damage has not been fully elucidated following cererbral ischaemia/prefusion.

The anti‐inflammatory effect of folic acid has been widely reported in various clinical conditions. For instance, folic acid supplementation mitigated Alzheimer's disease and improved cognitive function in Chinese elderly with MCI via lowering the levels of peripheral inflammatory cytokines.^11^ Guest et al observed an inverse association between cerebro‐spinal fluid (CSF) folate and CSF levels of IL‐6 in a healthy human cohort.^12^ Folic acid protects motor neurons against the increased homocysteine, inflammation and apoptosis in SOD1 G93A transgenic mice.^13^ Therefore, it is possible that folic acid deficiency (FD) would augment brain damage by influencing the inflammatory response in ischaemic brains.

Recent evidences indicated that Notch signalling participated in inflammatory response of activated microglia in cerebral ischaemia. Indeed, inhibition of Notch signalling reduced the cell numbers of activated microglia, decreased the expression of proinflammatory cytokines and the cerebral infarct size and improved functional outcome in a model of focal ischaemic stroke.^14^, ^15^ In addition, the transcriptional factor nuclear factor kappa B (NF‐κB) which is widely known as a key transcriptional factor is associated with the activation of microglia and the subsequent inflammatory responses following cerebral ischaemia.^16^ Cao et al showed that Notch‐1 and NF‐κB p65 signalling pathways operated in synergy in regulating the production of proinflammatory mediators in lipopolysaccharide (LPS)‐activated microglia.^17^ More specifically, Notch signalling can amplify the proinflammatory response of microglia by enhancing the NF‐κB p65 signalling.^18^ Thus, we hypothesized that folic acid modulates the production of proinflammatory cytokines in activated microglial cells by Notch‐1 and NF‐κB/p65 signalling pathways.

In this study, both rat MCAO model and in vitro oxygen‐glucose deprivation (OGD) BV2 cells were used to observe the effect of FD on activation of microglia, and investigate the impact of FD on the Notch‐1/NF‐κB p65 pathway. Additionally, it is well known that the CA1 region of hippocampus is unusually vulnerable to a variety of insults, including hypoxia‐ischaemia.^19^ Therefore, we examined the regional hippocampal sensitivity to ischaemic injury combined with FD.

## 2 METHODS

### 2.1 Animals

Thirty male Sprague‐Dawley rats weighing 200‐230 g (8 weeks old; Grade SPF, Certificate Number SCXK (Jing) 2012‐0001) were purchased from Peking Weitong Lihua Experimental Animal Technology Center (Beijing, China). The experimental protocols were approved by the Tianjin Medical University Animal Ethics Committee and performed in compliance with institutional guidelines under approved protocols. Rats were stratified according to body weight and randomized into three groups (20 per group): sham‐operated control group (SHAM), middle cerebral artery occlusion‐reperfusion group (MCAO), MCAO plus folic acid‐deficient diet group (MCAO + FD). In our previous study, compared with the concentration before intervention the rats fed with folic acid deficient deits (0.2 mg folic acid/kg; Keao Xieli Company, China) for 28 days significantly decreased in the folic acid concentration in serum.^20^ Therefore, in this study, the rats were pretreated with the normal (2.1 mg folic acid/kg) or folic acid‐deficient diets for 28 days prior to animal operation.

### 2.2 Surgical procedures

All rats were anaesthetized with 1% sodium pentobarbital (40 mg/kg) via intraperitoneal injection. The MCAO rats were induced by intraluminal filament technique as described previously.^21^ A head‐end spherical nylon thread was advanced by the left common carotid artery (MCA), through the left internal carotid artery and into the origin of the middle cerebral artery. The thread was pulled out 1 cm and cut off at 1 hour after the operation. Animals in SHAM group were treated by all procedures, except that the thread was not advanced to the origin of the MCA. The rats were then allowed to recover from anaesthesia at 37°C and were sacrificed at 24 hours after reperfusion for the following experiments.

A neurological score was assigned to each animal 10 minutes after waking up according to the Longa method.^21^ No deficit = 0; contralateral forelimb weakness = 1; circling to contralateral side = 2; partial paralysis on contralateral side = 3; and no spontaneous motor activity = 4. MCAO rats with neurological deficit scores of 1‐3 were left for the following experiments. The rats subjected to MCAO without any detectable neurological deficits or no spontaneous motor activity were excluded from the following investigations and analyses.

### 2.3 Haematoxylin and eosin staining

The brain tissues at 24 hours after the MCAO operation were processed for paraffin embedding and serial 6 μm sections were prepared (n = 4 per group). The sections were dewaxed in xylene and rehydrated in graded alcohols, then stained with haematoxylin and eosin (HE). Sequentially, the sections were dehydrated in alcohol gradients and xylene, then blocked by neutral gum. The pathological changes of brain tissues were observed by a light microscope (IX81; Olympus, Tokyo, Japan).

### 2.4 Fluoro‐Jade B staining

Fluoro‐Jade B (FJ‐B) staining was used to identify neuronal degeneration (n = 4 per group). The sections were dewaxed in xylene and rehydrated in graded alcohols, and then were immersed in a solution containing 1% sodium hydroxide in 80% alcohol for 5 minutes. This was followed by 2 minutes in 70% alcohol and 2 minutes in distilled water. The sections were then transferred to a solution of 0.06% potassium permanganate for 10 minutes, preferably on a shaker table to insure consistent background suppression between sections. The staining solution of FJ‐B (Millipore, Temecula, CA) was dropped onto the brain tissue. After 20 minutes, the slides were rinsed for one minute in each of three distilled water. Subsequently, the sections were placed on a warmer at 55°C for 5 minutes, cleared in xylene for 1 minute and examined with a light microscope (IX81; Olympus).

### 2.5 Cell culture and treatment

BV‐2 microglial cells were obtained from Tianjin Neurological Institute (Tianjin, China). The murine cell line BV2 was derived from primary microglial cell cultures and immortalized by infection with a v‐raf/v‐myc oncogene carrying retrovirus (J2).^22^ The cells were cultured in DMEM (4 mg folic acid/L; Sigma, St. Louis, MO) or folic acid‐deficient DMEM (0 mg folic acid/L; Sigma) supplemented with 10% foetal bovine serum (FBS) (Gibco, Gaithersburg, MD) for 7 days. N‐[N‐(3, 5‐difluorophenacetyl)‐l‐alanyl]‐S‐phenylglycine t‐butyl ester (DAPT; Sigma), a γ‐secretase enzyme inhibitor, was utilized to suppress the activation of Notch signalling.

The cells were divided into the control group, OGD group, oxygen glucose deprivation + DAPT (OGD + DAPT), oxygen glucose deprivation + folic acid deficiency (OGD + FD) and oxygen glucose deprivation + folic acid deficiency + DAPT group (OGD + FD + DAPT). N‐[N‐(3, 5‐difluorophenacetyl)‐l‐alanyl]‐S‐phenylglycine t‐butyl ester at a final concentration of 10 µmol/L was added in glucose‐free DMEM for OGD + DAPT group and OGD + FD + DAPT group 1 hour before hypoxia.^22^, ^23^, ^24^ The cells in the control group were maintained in DMEM supplemented with 10% FBS in an incubator with an atmosphere of 5% CO_2_ and 95% air at 37°C. To imitate the cerebral ischaemia/reperfusion model in vivo, the cells in the ODG group, OGD + DAPT group, OGD + FD group and OGD + FD + DAPT group were incubated in a three‐gas incubator at 37°C containing 1.0% O_2_ to initiate hypoxia, followed by 1 hour reoxygenation in a normoxia incubator. Finally, the cell samples were collected and kept in a −80°C freezer until further use.

### 2.6 Western blot analysis

Western blot was used to analyse protein expression in the BV‐2 cells and the hippocampus of the ipsilateral ischaemic hemisphere. The brain tissues or cells (n = 4 per group) were homogenized in RIPA buffer (20 mmol/L TRIS‐HCl pH 7.5, 150 mmol/L NaCl, 1 mmol/L EDTA, 1% Triton‐X100, 0.5% sodium deoxycholate, 1 mmol/L PMSF (Phenylmethanesulfonyl fluoride) and 10 µg/mL leupeptin; Beyotime Institute of Biotechnology, Shanghai, China) on ice for 30 minutes, then centrifuged at 15 000× g for 30 minutes at 4°C. The supernatants were collected and protein concentrations were determined by a Bicinchonininc acid (BCA) Protein Assay kit (Beyotime). Equal amounts of protein were separated by 10% sodium dodecyl sulphate‐polyacrylamide gel electrophoresis and transferred to polyvinyl indene difluoride membrane (PVDF; Millipore, Billerica, MA) and blocked with 5% BSA (Sigma) in 1× (Tris Buffered Saline‐Tween (TBST)20; pH 8.0) for 1 hour at room temperature. Subsequently, the membranes were incubated with mouse anti‐IL‐6 (1:1000; Abcam, Cambridge, MA), rabbit anti‐TNF‐α (1:1000; Abcam), rabbit anti‐IL‐1β (1:1000; Abcam), rabbit anti‐Notch1 (1:1000; Cell Signaling Technology [CST], Danvers, MA), rabbit anti‐NF‐κB p65 (1:1000; CST)，rabbit anti‐IkBα (1:1000; CST), rabbit anti‐pIkBα (1:1000; CST) and mouse anti‐β‐actin (1:2000; CST) primary antibodies overnight at 4°C. They were then incubated with the secondary antibodies (Horseradish peroxidase (HRP)‐linked anti‐rabbit IgG; HRP‐linked anti‐mouse IgG; 1:2000; CST) for 1 hour at room temperature. Then, the proteins were detected by chemiluminescence reagents (Millipore) and observed using a ChemiDocTM XRS + Imaging System (Bio‐Rad, Hercules, CA). The protein levels were quantified by densitometry using Image j 1.4.3.67.

### 2.7 Immunofluorescence

The slides of brain sections (n = 4 per group) were fixed in 4% paraformaldehyde, disposed in 3% H_2_o_2_ for 10 minutes at room temperature, repaired by citric acid antigen and then blocked with goat serum for 1 hour at 37°C. The sections were incubated overnight at 4°C with the primary antibodies (mouse anti‐IL‐6, rabbit anti‐TNF‐α, rabbit anti‐IL‐1β, mouse anti‐Iba‐1, 1:200, Abcam; rabbit anti‐Notch1, rabbit anti‐ NF‐κB p65, 1:200, CST; rabbit anti‐Iba‐1, 1:1000, Wako, Chuo‐Ku, Japan). Thereafter, the sections were washed in PBS and then incubated with the FITC (Fluorescein)‐conjugated goat anti‐rabbit or TRITC (Rhodamine)‐conjugated goat anti‐mouse secondary antibodies (1:100; Zhongshan Goldbridge Biotechnology, Beijing, China) for 1 hour at room temperature. The nucleus was stained by 4, 6‐diamidino‐2‐phenylindole (DAPI; Solarbio, Beijing, China) before 10 minutes of mounting.

BV‐2 cells seeded on the cover slips were fixed in 4% paraformaldehyde for 30 minutes, and then blocked with goat serum for 1 hour at room temperature. The cover slips were incubated with the primary antibodies (rabbit anti‐NF‐κB p65, 1:200, CST) at 4°C overnight. After rinsing in PBS for three times, the cover slips were incubated with the FITC‐conjugated goat anti‐rabbit antibody (1:100) at 37°C for 1 hour. Finally, the cover slips were incubated with DAPI for 10 minutes and mounted with a fluorescent mounting medium. The positive cells were observed with a fluorescence microscope (IX81; Olympus) and analysed by Image Pro Plus 6.0 software.

### 2.8 Statistical analysis

All data were analysed by spss v. 20.0 and expressed as mean ± SD ($\overset{-}{x}\pm SD$). Differences between means were determined by one‐way ANOVA followed by Student‐Newman‐Keuls multiple range tests. *P* < 0.05 was considered statistically significant.

## 3 RESULTS

### 3.1 Folic acid deficiency induced neural cell injury in hippocampal subregions of the ipsilateral ischaemic hemisphere

**Figure 1:**
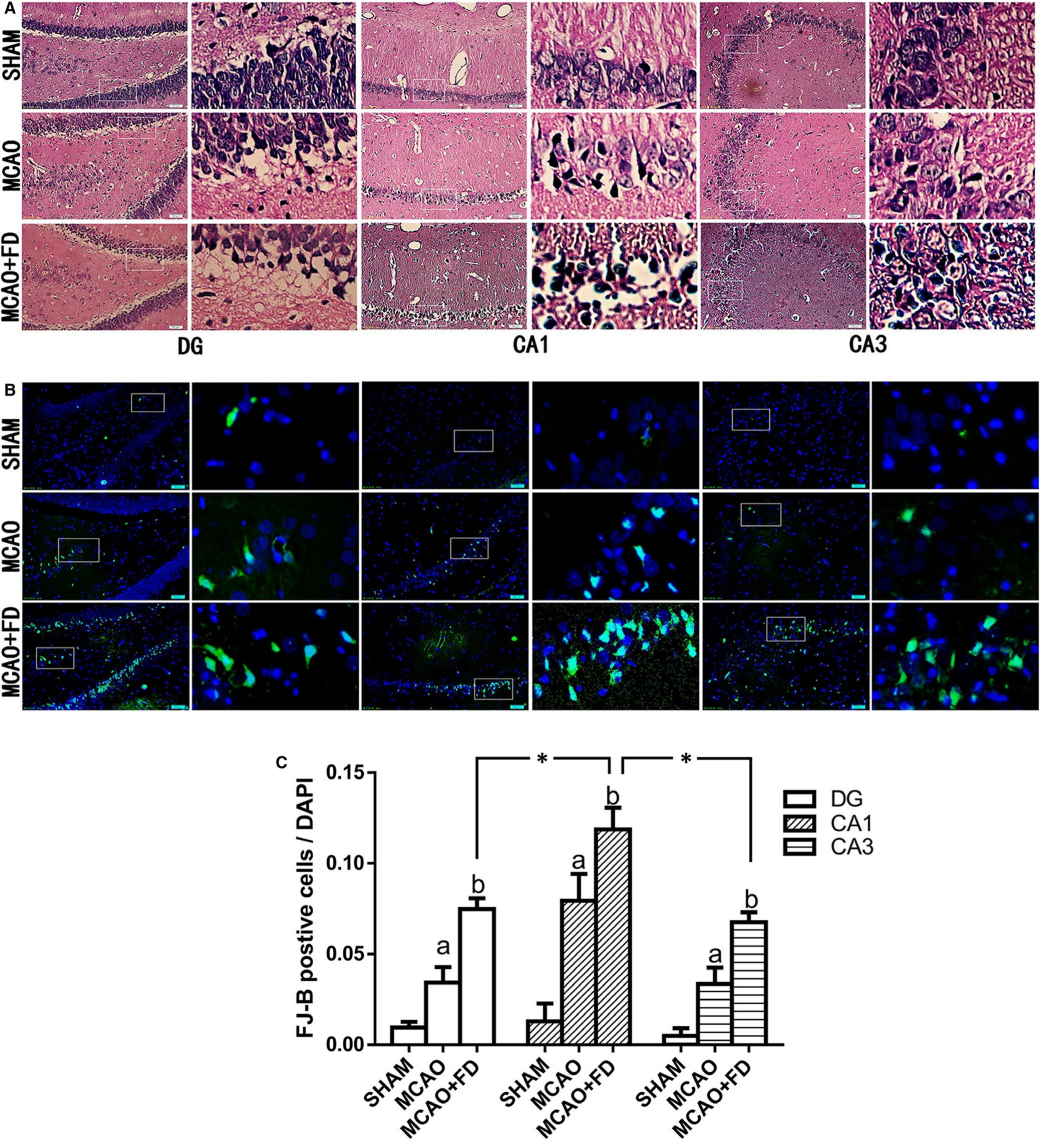
Folic acid deficiency (FD) induced neural cell injury in hippocampal subregions of the ipsilateral ischaemic hemisphere. A, Histological outcomes of haematoxylin and eosin staining of hippocampus (CA1, CA3, and DG regions). B, Photomicrographs of neuronal degeneration by Fluoro‐Jade B (FJ‐B) (green) assay. Each right‐hand column depicts a magnified image of the rectangular region of the corresponding image in the left column. C, The ratio of FJ‐B‐positive cells and 4, 6‐diamidino‐2‐phenylindole (DAPI)‐positive cells (total cells) in hippocampus DG, CA1 and CA3 regions. The data are presented as the mean ± SD (n = 4 per group, four sections and four fields per section were chosen for analysis in each rat). *P < 0.05 vs the CA1 region, a *P* < 0.05 vs the sham‐operated control group (SHAM), b *P* < 0.05 vs the MCAO group. Scale bars = 50 μm

The morphology of neural cells from the hippocampal CA1, CA3 and DG regions was observed by HE staining after 24 hours ischaemia‐reperfusion. In the SHAM group, the neurons were observed with clear round outline, well‐preserved cytoplasm and distinct integrated nucleus, while some neurons in the MCAO group were arranged disorderly and appeared indistinct, lacking a clear cell boundary, with a pyknotic or severely shrunken nucleus. Compared with the MCAO group, the cell damage was further manifested in MCAO + FD group (Figure 1A).

Next, neuronal degeneration in three subregions of hippocampus was detected by the FJ‐B staining after 24 hours ischaemia‐reperfusion. Compared to the SHAM group, the relative number of the FJ‐B positive cells (the ratio of FJ‐B positive cells to DAPI‐positive cells) in CA1, CA3 and DG regions of hippocampus significantly increased in MCAO group (*P* < 0.05). Moreover, a further increase in the relative number of the degenerating cells was observed after FD treatment, as evidenced by more positive cells of FJ‐B staining than those of the MCAO group (*P* < 0.05, Figure 1B).

Additionally, we also investigated the regional differences of neuronal vulnerability within the ischaemic hippocampus after FD treatment (Figure 1C). The results showed that the most heavily damaged area was the subfield of CA1 after FD treatment. The CA3 and DG areas had also undergone degeneration, but to a lesser extent compared to CA1 subfield. No significant difference was observed between the CA3 and DG hippocampal subregions.

### 3.2 Folic acid deficiency raised MCAO‐induced microglial activation

**Figure 2:**
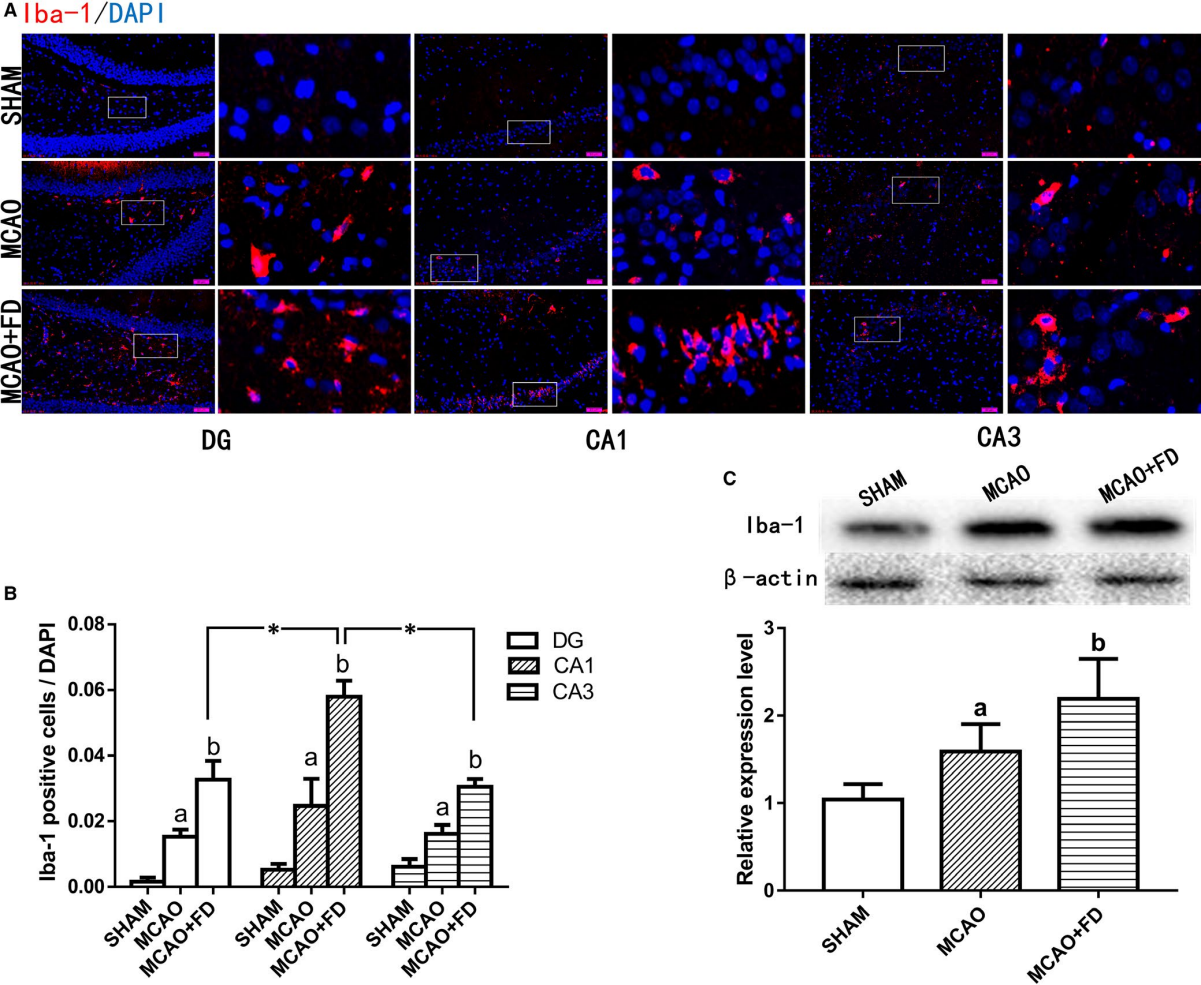
Folic acid deficiency (FD) induced activation of microglia in hippocampus following ischaemia‐reperfusion. A, Immunofluorescence analysis of Iba‐1 (red, a microglia marker) in hippocampus CA1, CA3 and DG regions. Nuclei are stained with 4, 6‐diamidino‐2‐phenylindole (DAPI) (blue). Each right‐hand column depicts a magnified image of the rectangular region of the corresponding image in the left column. B, Quantification of Iba‐1‐positive cells/total number of DAPI‐stained nuclei in hippocampus subregions. C, Western Blot analysis of Iba‐1 in the hippocampus extracts. β‐actin protein was used here as an internal control. The data are presented as the mean ± SD (n = 4 each group). *P < 0.05 vs the CA1 region, a *P* < 0.05 vs the sham‐operated control group (SHAM), b *P* < 0.05 vs the MCAO group. Scale bars = 50 μm

To test whether neural cell injury caused by FD is associated with microglial activity, the immunostaining for Iba‐1, a microglia specific marker, was performed to detect the effect of FD on microglia activation following cerebral ischaemia. As shown in Figure 2, there were only few Iba‐1‐immunoreactive cells in the SHAM group. The strikingly increased number of Iba‐1 positive (Iba‐1+) cells was observed in the MCAO group, which was further raised by FD treatment in all three hippocampus subregions. Additionally, compared with CA3 and DG regions, the change in the number of Iba‐1 positive cells was the greatest in CA1 from FD‐treated ischaemic brains (*P* < 0.05).

Western blot analysis of hippocampus extracts further confirmed that the Iba‐1 protein express was significantly increased in FD + MCAO group, compared with that in MCAO group (Figure 2; *P* < 0.05).

### 3.3 Folic acid deficiency induced neuroinflammatory responses in activated microglia following ischaemia/reperfusion injury

**Figure 3:**
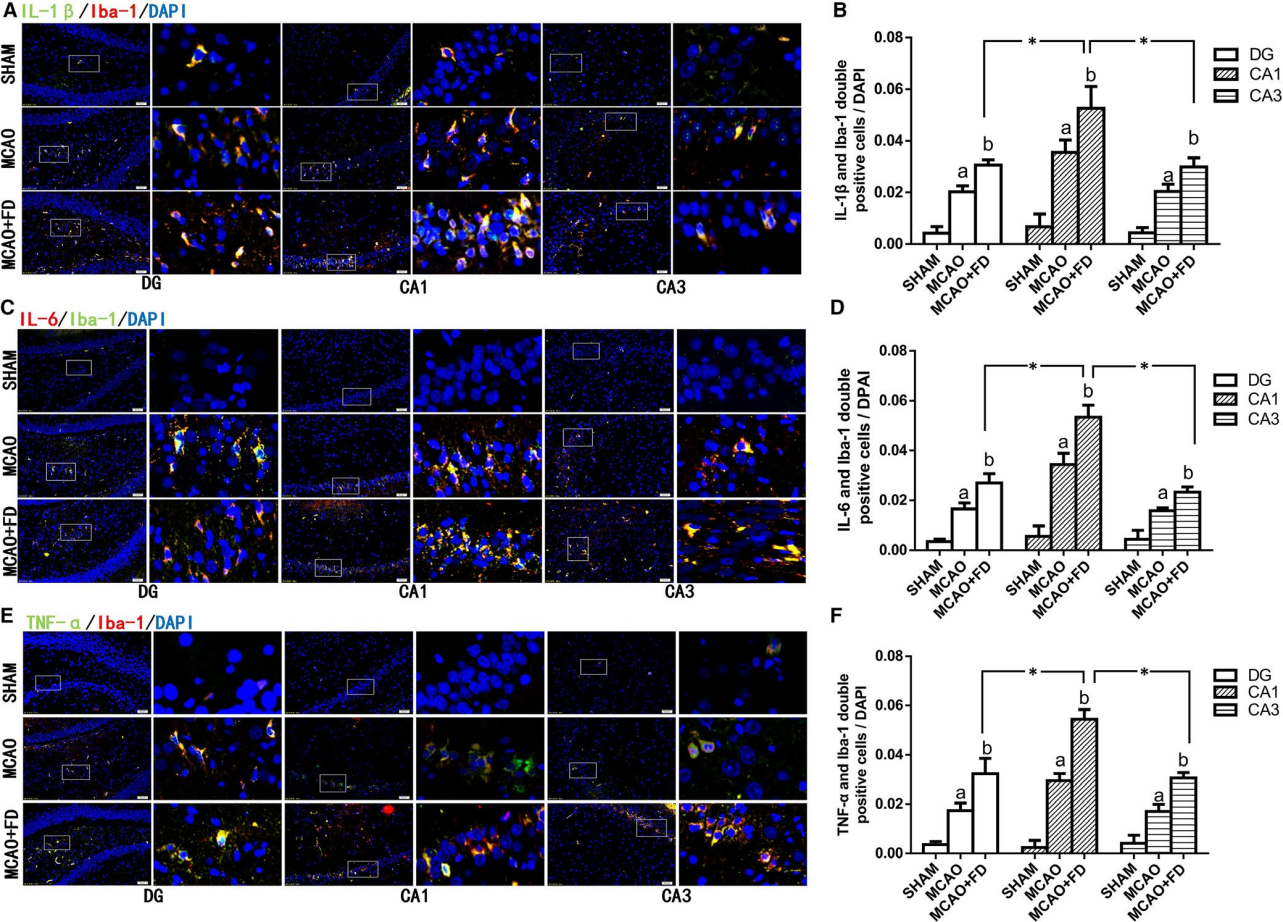
Folic acid deficiency (FD) induced neuroinflammatory cytokines accumulation in activated microglia following ischaemia reperfusion. A, Dual‐immunofluorescence of Iba‐1(red) and IL‐1β (blue) in hippocampal subregions. C, Dual‐immunofluorescence of Iba‐1(blue) and IL‐6 (red) in hippocampal subregions. E, Dualimmunofluorescence of Iba‐1(red) and TNF‐α (blue) in hippocampal subregions. Each right‐hand column depicts a magnified image of the rectangular region of the corresponding image in the left column. Nuclei were stained for 4, 6‐diamidino‐2‐phenylindole (DAPI) (blue). Quantification of Iba‐1+/IL‐1β+(E), Iba‐1+/IL‐6+ (D) and Iba‐1+/TNF‐α+ (F) double‐stained cells/total number of DAPI‐stained nuclei in hippocampal CA1, CA3 and DG regions. The data are presented as the mean ± SD (n = 4 each group). *P < 0.05 vs the CA1 region, a *P* < 0.05 vs the sham‐operated control group (SHAM), b *P* < 0.05 vs the MCAO group. Scale bars = 50 μm. IL‐1β, interleukin‐1beta; IL‐6, interleukin‐6; TNF‐α, tumour necrosis factor‐α

To investigate whether neuroinflammatory cytokines located in microglia were capable of responding to FD, we assessed the colocalization of IL‐1β (IL‐6 or TNF‐α) immunoreactivity with the microglia marker Iba‐1 in hippocampal CA1, CA3 and DG regions at 24 hours after cerebral ischaemia. As shown in Figure 3, most Iba‐1+ cells were also IL‐1β, IL‐6 or TNF‐α‐positive in all three regions of hippocampus examined. Ischaemic stroke induced massive production of TNF‐α, IL‐1β and IL‐6 in Iba‐1+ cells. After FD treatment, the expression of three neuroinflammatroy cytokines was further raised in Iba‐1+ cells, compared to the levels in the MCAO group (Figure 3A,C,E).

In addition, although the increase in co‐expression of Iba‐1 and TNF‐α (IL‐1β or IL‐6) was statistically significant in both CA3 and DG regions of the hippocampus in the MCAO + FD group, compared with the MCAO group, the highest levels of the inflammatory cytokines were observed in hippocampal area CA1 (Figure 3B,D,F).

### 3.4 Folic acid deficiency increased Notch‐1 and NF‐κB p65 protein expression following ischaemic injury

**Figure 4:**
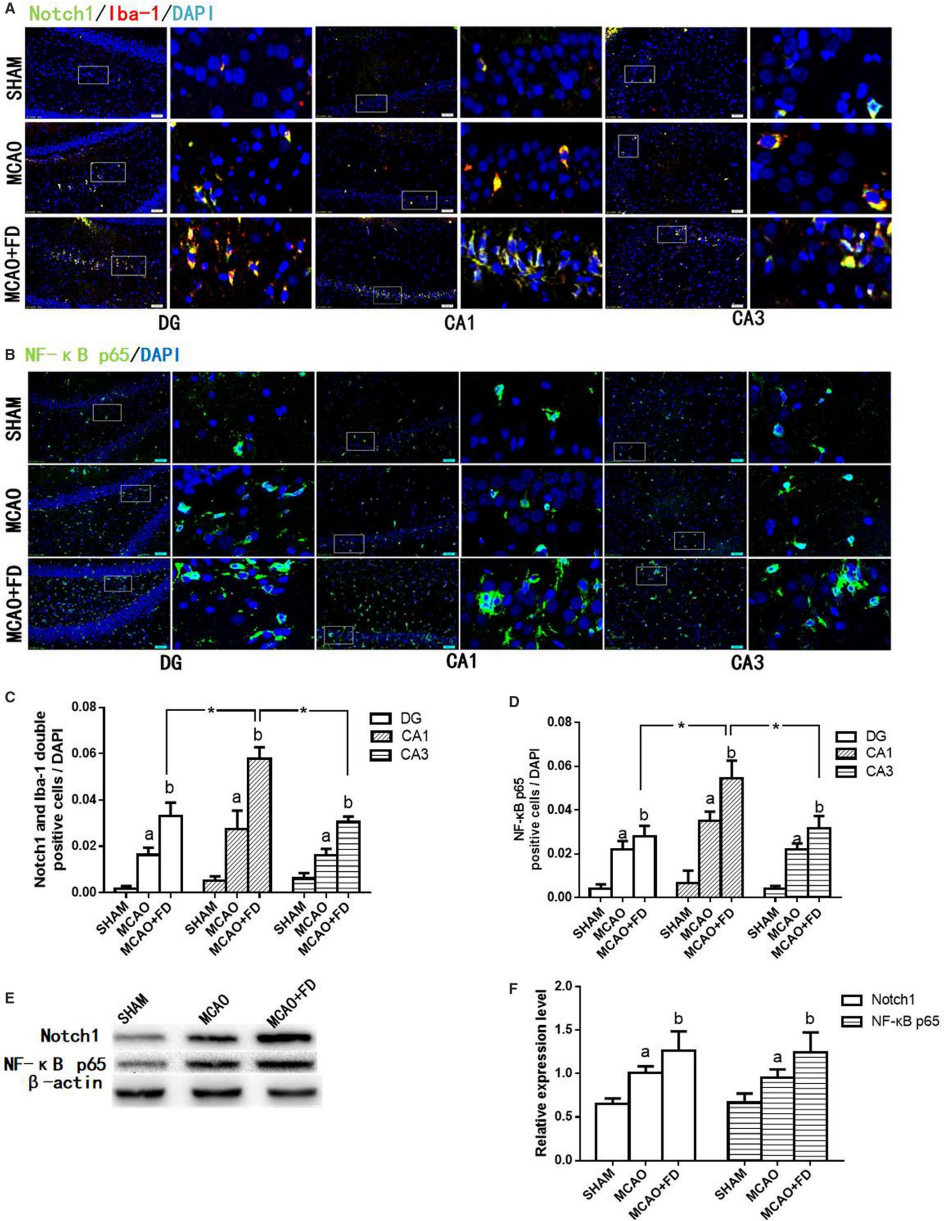
Folic acid deficiency (FD) increased Notch‐1 and nuclear factor kappa B (NF‐κB) p65 expression following ischaemia‐reperfusion. A, Co‐staining of Iba‐1 (red) and Notch1 (green) in hippocampal subregions by double immunofluorescence. B, Single stained of NF‐κB p65 (green) in hippocampal subregions by double immunofluorescence. C, Quantification of the number of Iba‐1 and Notch1 double positive cells/total number of 4, 6‐diamidino‐2‐phenylindole (DAPI)‐stained nuclei in hippocampus subregions. D, Quantification of the number of NF‐κB p65‐positive cells/total number of DAPI‐stained nuclei in hippocampus subregions. E, Western blot analysis of Notch1 and NF‐κB p65 in hippocampus protein extracts. F, The levels of Notch1 and NF‐κB p65 proteins were quantified and normalized to ß‐actin levels. The data are presented as the mean ± SD (n = 4 each group). *P < 0.05 vs the CA1 region, a *P* < 0.05 vs the sham‐operated control group (SHAM), b *P* < 0.05 vs the MCAO group

Notch‐1 and NF‐κB p65 have been proven to synergistically modulate the proinflammatory function in activated microglia. More specifically, Notch signalling can amplify the proinflammatory response of microglia by enhancing the NF‐κB p65 signalling.^25^ To clarify the molecular mechanism underlying FD‐induced neuroinflammation response in activated microglia, the protein expression of Notch1 was investigated in Iba‐1‐labelled cells in three hippocampus subregions 24 hours after ischaemia reperfusion. The results from immunofluorescence showed that Notch1 is expressed in microglia and up‐regulated after cerebral ischaemia. FD further raised the MCAO‐induced expression of Notch1 protein in activated microglia in three hippocampus subregions. Additionally, after FD treatment, the highest level of Notch1 expression was observed in hippocampal area CA1. However, moderate Notch1 expression was also observed in the DG and CA3 region (Figure 4A,C). The same trend was also seen for NF‐κB p65 expression level in all experiment groups (Figure 4B,D).

Consistent with the immunohistochemistry, Western blot analysis showed that the protein expression levels of Notch1 and NF‐κB p65 were significantly augmented in hippocampus protein extracts after FD treatment (*P* < 0.05, Figure 4E,F).

### 3.5 Folic acid deficiency promoted the production of neuroinflammatory cytokines and Notch1/NF‐κB protein expression in BV‐2 cells exposed to oxygen‐glucose deprivation

**Figure 5:**
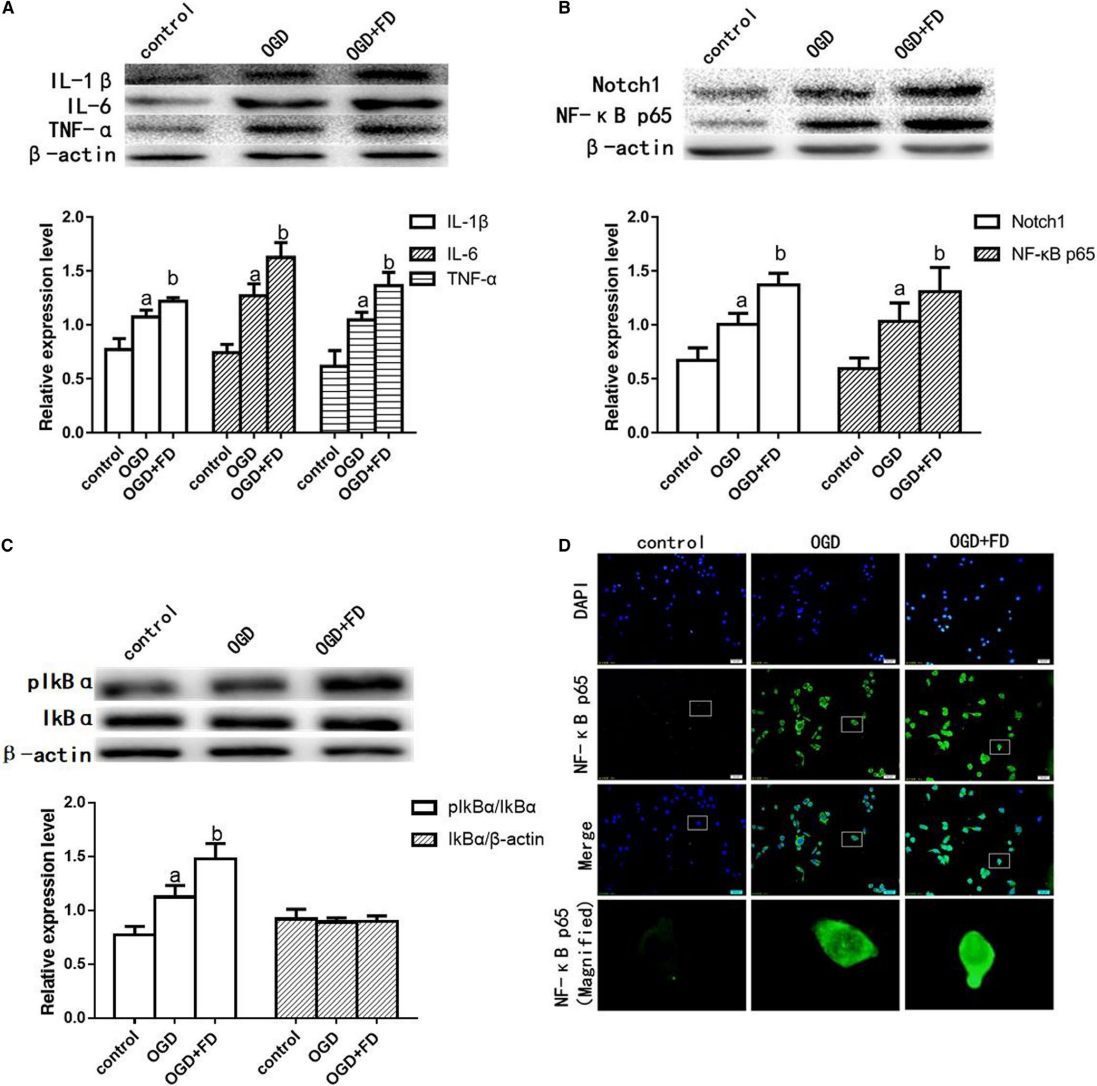
Oxygen‐glucose deprivation (OGD) activated Notch1/nuclear factor kappa B (NF‐κB) and neuroinflammatory cytokines protein expression in BV‐2 cells. A, Western blot analysis of interleukin‐1beta (IL‐1β), interleukin‐6 (IL‐6) and tumour necrosis factor‐α (TNF‐α); (B) Western blot analysis of Notch1 and NF‐κB p65. C, Western Blot analysis of pIkBα; β‐actin protein was used here as an internal control. D, Nuclear factor kappa B p65 immunostaining of the BV‐2 cells. The data are presented as the mean ± SD (n = 4 each group). a *P* < 0.05 vs the control group, b *P* < 0.05 vs the OGD group

Next, we used BV‐2 cells to further investigate whether FD promoted OGD‐induced production of proinflammatory cytokines in microglia. Consistent with our in vivo results, western blot results showed a similar increase in protein expression of IL‐6, IL‐1β and TNF‐α in OGD group, compared with the control group (*P* < 0.05; Figure 5A). FD significantly raised the expression of three inflammatory mediators in OGD‐induced BV‐2 cells. Furthermore, it has also been validated in BV‐2 cells that the protein expression of Notch1, pIκBα and NF‐κB p65 increased significantly after OGD, compared with the control group, and FD exacerbated this trend (*P* < 0.05; Figure 5B,C).

Under control conditions, inactive NF‐κB is localized to the cytoplasm by its interaction with inhibitory IκB proteins such as IκBα. Dissociation from IκB and subsequent nuclear translocation of NF‐κB is initiated by the degradation of IκB through cytokine‐induced IkB kinase activation.^26^ Once activated, NF‐κB acts as an important transcription regulator for the expression of various genes involved in inflammation, infection and immune response including the genes for IL‐1β, TNF‐α and IL‐6. Therefore, the occurrence of activated nuclear NF‐κB p65 was investigated by immunohistochemistry (Figure 5D). We found that the OGD‐exposed cells showed nuclear localization of NF‐κB p65. More intense NF‐κB P65 label indicative of cell activation localized to the nucleus of FD‐treated OGD cells. By contrast, only sparse and cytosolic (ie inactive) NF‐κB p65 labelling was visible in normal untreated cells.

### 3.6 Inhibition of Notch signalling by DAPT partly reversed FD‐induced expression of neuroinflammatory cytokines in BV‐2 cells with OGD exposure

**Figure 6:**
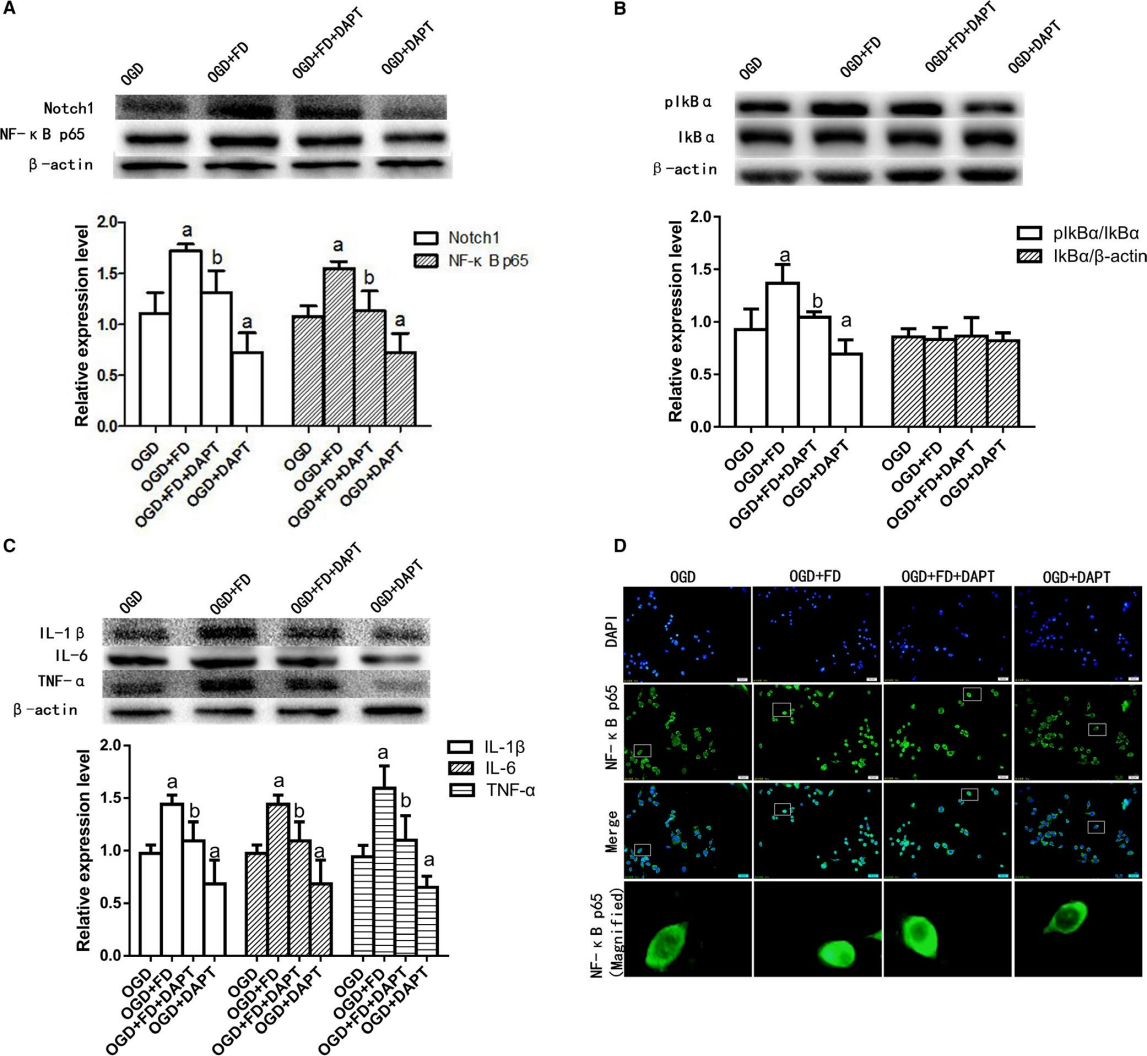
N‐[(3, 5‐Difluorophenyl) acetyl]‐L‐alanyl‐2‐ phenylglycine‐1, 1‐dimethylethyl ester (DAPT) inhibited the activation of the Notch1/NF‐κB and neuroinflammatory cytokines caused by folic acid deficiency (FD) and oxygen‐glucose deprivation (OGD). A, Western blot analysis of Notch1 and nuclear factor kappa B (NF‐κB) p65. B, Western blot analysis of pIkBα. C, Western blot analysis of interleukin‐1beta (IL‐1β), interleukin‐6 (IL‐6) and tumour necrosis factor‐α (TNF‐α). β‐actin protein was used here as an internal control. D, Nuclear factor kappa B p65 immunostaining of the BV‐2 cells. The data are presented as the mean ± SD (n = 4 each group). a *P* < 0.05 vs the OGD group, b *P* < 0.05 vs the OGD+FD group

To further clarify the role of Notch signalling in microglial activation by FD, BV‐2 cell cultures were challenged with OGD and FD in the presence or absence of DAPT. N‐[N‐(3, 5‐difluorophenacetyl)‐l‐alanyl]‐S‐phenylglycine t‐butyl ester is a γ‐secretase enzyme inhibitor that inhibits Notch1 protein expression. Compared with OGD + FD group, the expression of Notch1, pIkBα and NF‐κB p65 decreased significantly in OGD + FD + DAPT group (*P* < 0.05; Figure 6A‐C). In addition, FD‐induced up‐regulation of the pro‐inflammatory cytokines IL‐1β, IL‐6 or TNF‐α was markedly diminished by DAPT (Figure 6C).

The immunostaining exhibited that DAPT reversed NF‐κB p65 translocation from the cytoplasm to the nucleus induced by FD (Figure 6D). It was suggested that DAPT may attenuate microglia‐mediated neuroinflammation by modulating the Notch1/NF‐κB pathway in the in vitro ischaemia condition.

## 4 DISCUSSION

Stroke is a leading cause of death and permanent adult disability all over the world and remains a major challenge to public health.^27^ Folate deficiency increases brain damage following cerebral ischaemia‐reperfusion.^9^ In this study, we showed that FD induced significant cell injury and microglial activation in hippocampus after cerebral ischaemia‐reperfusion. It was for the first time validated in microglial cultures exposed to OGD and rat MCAO model that there was the relationship between FD and microglial‐induced neuroinflammation, which may occur via Notch/NF‐κB p65 pathway regulation.

Folate is essential for brain development and function. A meta‐analysis demonstrated a significant benefit of folic acid supplement in preventing stroke in countries without mandatory folic acid food fortification.^28^ Furthermore, in a recent study on Chinese patients with hypertension, folic acid intervention could reduce the risk of stroke.^29^ In animal studies, folic acid supplementation would reduce brain injury and improve neurological outcome in a neonatal piglet model of traumatic brain injury.^30^ Folate deficiency and elevated homocysteine levels increase the vulnerability of cultured neurons partly via mechanisms related to uracil misincorporation, oxidative DNA damage and impaired DNA.^31^ Consistent with previous studies, our present data showed that FD aggravated neuron damage in hippocampal subregions following ischaemia‐reperfusion. It suggested that folate‐rich dietary intervention may have beneficial effects on functional recovery and therefore therapeutic potential against ischaemic stroke.^32^

Microglia are regarded as the first responders and the principal immune cells in the central nervous system that mediate neuroinflammation following cerebral ischaemia.^33^ Increasing evidences suggest that activated microglial cells can act as double‐edged swords in ischaemic stroke. The mild or moderate activation of microglial cells migrate to the ischaemic area to clear the harmful agents and maintain tissue homoeostasis.^34^ However, uncontrolled or over‐activated microglia may exacerbate tissue damage and neuronal death by producing excessive inflammatory cytokines, chemokines and oxygen/nitrogen‐free radicals, such as NO, TNF‐α, IL‐1β, IL‐6 and reactive oxygen species (ROS).^35^, ^36^ Lambertsen et al proved that microglia macrophages, especially activated microglia, were the predominant source of TNF‐α after induction of pMCAO.^37^ The cells that expressed IL‐1β had the morphologic features of microglia and macrophages in permanent unilateral occlusion of the middle cerebral artery.^38^ Meanwhile, in the permanent rat MCAO model, the activated microglia were thought of an important source of IL‐6.^39^ Taken together, regulating the microglia activity may be helpful for recovery from ischaemic stroke. In our models, FD treatment resulted in the over‐activation of microglial cells and the high expression of neuroinflammatory factors, which may further induce cell injury. So the hyperactivation of microglial cells caused by FD appeared to be harmful for the ischaemic brain. Thus, our results also suggested that appropriate activation of microglial cells may be key to promote stroke recovery.

Recent evidence indicated that classical Notch signalling was activated in microglial cells in vitro and vivo following hypoxic exposure.^40^ Activated Notch pathway promotes microglial production of proinflammatory mediators that contribute to neuronal damage.^41^ Notch signalling was further identified to regulate microglial activation via NF‐κB pathway after hypoxic exposure.^42^ In the cytosol, the canonical mechanism of NF‐κB activation involves phosphorylation of the inhibitory IκB subunit by the IκB kinase complex, especially the inhibitory protein IκBα.^43^ Some studies suggested that the basal IκBα expression was under the control of the various components of the CBF1/Notch signal transduction pathway.^44^, ^45^ This further demonstrated that the Notch and NF‐κB pathways operate synergistically in regulating the production of proinflammatory mediators in activated microglia. Antisense Notch mice, when injected with LPS or subjected to MCAO, produced less IL‐1β and TNF‐α and also had attenuated NF‐κB p65 activity, indicating that Notch signalling may play a role in microglia toxicity after ischaemia.^46^, ^47^ Pretreatment of microglia with GSI（a γ‐ secretase inhibitors） substantially reduces NF‐κB p65 nuclear translocation, coupled with a decrease in microglia proliferation and inflammation‐related cytokines that play a crucial role in mediating neurotoxicity.^48^ In our study, we found that Notch1 and NF‐κB p65 protein expression increased after FD combined with ischaemia‐reperfusion injury. In vitro, blocking of Notch1 with DAPT in activated BV‐2 microglia not only markedly suppressed Notch1 protein expression but also inhibited the NF‐κB p65 nuclear translocation and the expression of the phosphorylation of IkBα. Meanwhile, FD‐induced increase in the expression of IL‐1β, IL‐6 and TNF‐α was also sensitive to the Notch1 inhibition by DAPT. These data provided evidence that the Notch1/NF‐κB p65 pathway might be a molecular mechanism underlying FD‐induced microglial activation and neuroinflammatory injury in MCAO rats.

We previously reported that folic acid supplementation stimulated neural stem cell proliferation by Notch signalling after rat experimental stroke;^49^ however, this study showed that FD enhanced Notch1 expression in activated microglia. This suggested that folic acid regulated Notch1 expression in a different way in two types of neural cells types in rat ischaemic brain.

The hippocampus is well known to be one of the brain regions most vulnerable to hypoxia/ischaemia. Studies have shown that the vulnerability of neurons to cerebral ischaemia varies widely among different subregions in the hippocampus. CA1 pyramidal neurons in hippocampus are particularly vulnerable to ischaemic insult, whereas CA3 neurons are relatively resistant.^50^, ^51^, ^52^ Similar with hypoxia stimuli, the present study showed that the CA1 region of the hippocampus was also most vulnerable to FD stress. However, regional susceptibility of hippocampus may be different to different external stimuli or stress. For instance, CA3 was found to be more vulnerable than CA1 in experimental models of controlled cortical impact‐induced brain injury.^53^

Furthermore, our study exhibited that the inflammatory factors were induced not only in microglia of the CA1 region in the ischaemic hippocampus but also of the CA3 and DG regions. DG and CA3 rat expressed similar levels of the inflammatory factors, while CA1 rat expressed much more. It suggested that the inflammatory response of the CA1 region of the hippocampus was particularly vulnerable to ischaemic injury and FD treatment. Differential responses of microglial neuroinflammation caused by FD and MCAO may underlie differential vulnerability of neural cells among CA1, CA3 and DG.

In conclusion, the present study showed that combination of hypoxia‐ischaemia and FD caused more severe neural cell injury and microglial inflammatory response in different hippocampus subregions, compared to hypoxia‐ischaemia alone. Inflammatory factor activation and up‐regulated expression of Notch1, pIkBα and NF‐κB p65 were attenuated by DAPT in FD microglial cell cultures exposed to hypoxia. FD may worsen neural cell injury and enhance the expression of inflammatory mediators following brain hypoxia‐ischaemia through the Notch signalling which operates synergistically with NF‐κB pathway in activated microglia. The results of this study suggested that folic acid supplementation might be a therapeutic strategy to alleviate various microglia‐mediated neuroinflammation by inhibition of microglial overactivation or targeting Notch1/NF‐κB p65 pathway.

## CONFLICT OF INTEREST

The authors declare that they have no competing interests.

## AUTHOR CONTRIBUTIONS

The authors' responsibilities were as follows. Xumei Zhang, Guowei Huang and Man Cheng designed the research; Man Cheng, Liu Yang, Zhiping Dong, Mengying Wang, Yan Sun, Huan Liu, Xuan Wang and Na Sai conducted the experiments and analysed the data; Xumei Zhang and Man Cheng wrote the manuscript; and all authors read and approved the final manuscript.
